# Regulation of sexually dimorphic placental adaptation in LPS exposure-induced intrauterine growth restriction

**DOI:** 10.1186/s10020-023-00688-5

**Published:** 2023-09-18

**Authors:** Da Som Jeong, Ji-Yeon Lee, Myoung Hee Kim, Ji Hoon Oh

**Affiliations:** 1 Department of Anatomy, Embryology Laboratory, Yonsei University College of Medicine, Seoul, 03722 Republic of Korea; 2 Present Address: Department of Internal Medicine, Yonsei University College of Medicine, Seoul, 03722 Republic of Korea; 3 Vivozon, Inc, Kolon Digital Tower3, 49, Achasan-ro, Seongdong-gu, Seoul, Republic of Korea; 4 Department of Biological Sciences, Keimyung University College of Natural Sciences, Daegu, 42601 Republic of Korea

**Keywords:** Sexual dimorphism, Placental adaptation, Prenatal maternal stress, Placenta transcriptome, Intrauterine growth restriction

## Abstract

**Background:**

Sexual dimorphism in placental physiology affects the functionality of placental adaptation during adverse pregnancy. Defects of placental function compromise fetal programming, affecting the offspring’s adult life. However, studies focusing on the relationship between sex-specific placental adaptation and consequent fetal maldevelopment under sub-optimal uterus milieu are still elusive.

**Methods:**

Here, we investigated the effects of maternal lipopolysaccharide (LPS) exposure between placental sex. Pregnant ICR mice received intraperitoneal injection of phosphate-buffered saline or 100, 200, and 400 µg/kg LPS on the gestational day (GD) 15.5. To determine whether prenatal maternal LPS exposure resulted in complicated pregnancy outcomes, survival rate of embryos was calculated and the growth of embryos and placentas was examined. To elucidate global transcriptomic changes occurring in the placenta, total RNA-sequencing (RNA-seq) was performed in female and male placentas.

**Results:**

LPS administration induced placental inflammation in both sexes at GD 17.5. Prenatal infection resulted in growth retardation in both sexes of embryos, and especially more prevalently in male. Impaired placental development was observed in a sex-specific manner. LPS 400 µg/kg reduced the percentage area of the labyrinth in females and junctional zone in males, respectively. RNA-sequencing revealed widespread sexually dimorphic transcriptional changes in placenta. In particular, representative changes were involved in biological processes such as trophoblast differentiation, nutrient/ion transporter, pregnancy, and immune system.

**Conclusions:**

Our results present the sexually dimorphic responses of placental physiology in intrauterine growth restriction model and provide tentative relationship further to be elucidated between sex-biased placental functional change and long-term effects on the offspring’s later life.

**Supplementary Information:**

The online version contains supplementary material available at 10.1186/s10020-023-00688-5.

## Background

Maternal pathogenic infection during pregnancy is an increasing risk factor for abnormal pregnancy. Prenatal stress can induce diverse modifications in biological processes and physiological and metabolic functions, predisposing to complicated pregnancy outcomes, including reduced birth weight, metabolic and cardiac diseases, and neurodevelopmental disorders. Experiments in animal models have shown that stresses exerted during pregnancy increase glucocorticoid levels, which stimulate the offspring’s hypothalamic–pituitary–adrenal axis and cause several developmental diseases such as schizophrenia, autism spectrum disorder (ASD), and behavioral and cognitive impairments (Seckl 2004; Gore et al. 2014). External stimuli can induce a variety of adversities that alter early-life fetal programming as well as developmental stage, which can eventually affect the offspring’s health later in life (Glover et al. 2018; McGowan and Matthews 2018).

Sexual dimorphism is a phenomenon in which the two sexes of the same species display different characteristics beyond the differences in their sexual organs. Most of the sex differences in adult diseases are usually explained by hormones and sex chromosomes (Arnold et al. 2017; Bramble et al. 2017). Recently, the concept of “fetal origin” has received attention for providing another important perspective on sexual dimorphism (Bermejo-Alvarez et al. 2011; Gilbert and Nijland 2008). The developmental origins of health and disease hypothesis highlighted this concept by emphasizing the effect of epigenetic and environmental causes occurring during development on sexual dimorphism in diseases that arise during adulthood (Heindel and Vandenberg 2015). Animal models provided evidences that prenatal stimuli-induced disorders, including mental disorders, impaired cognition, and ASD, are caused by sex-specific regulation of epigenetic modifications (Natt et al. 2017; Bale 2011; Schneider et al. 2016; Lussier et al. 2021).

As a maternal–fetal interface, the placenta plays pivotal roles in the regulation of normal fetal development by orchestrating the crosstalk between the maternal and fetal environments. For immune tolerance, the placenta counters the maternal immune response to protect the fetus, which is foreign to the mother (Kanellopoulos-Langevin et al. 2003). Moreover, the placenta can adapt to the *in utero* environment in response to even fine stimuli to ensure appropriate fetal growth, which is called placental adaptation (Hayward et al. 2016). Morphological changes can be indicative of functional adaptation: the surface area, the thickness of the placental barrier, and the architectural arrangements of the placental vasculature for the exchange of materials between the maternal and fetal environment (Zhang et al. 2015). Placental dysfunction is shown to be closely related to gestational complications such as intrauterine growth restriction (IUGR), preterm birth, preeclampsia (PE), miscarriage, and gestational diabetes (Longtine and Nelson 2011; Burton and Jauniaux 2018).

Human studies have profiled healthy human female and male placentas, highlighting sexually dimorphic expressions in the regulation of metabolism, chromatin modification, and splicing (Nugent and Bale 2015; Gonzalez et al. 2018). Gender differences in growth, metabolic regulation, and epigenetic modulations of placenta provide potential differences in the early-life environment in both sexes (Sood et al. 2006; Buckberry et al. 2014). Epidemiological studies have demonstrated sex-specific placental functions in a disturbed maternal immune system, suggesting that female and male fetuses may cope with different mechanisms. However, the mechanisms underlying the regulation of sex-specific adaptation under adverse conditions are not well understood.

To explore the sex-specific placental adaptation accompanied by impaired intrauterine growth, we used an animal model by administering lipopolysaccharide (LPS) to pregnant ICR mice on the gestational day (GD) 15.5. To investigate the prevalent sexual dimorphism of the placenta in the current model where IUGR was induced, we analyzed morphological changes using the histology and examined placenta transcriptome to identify the differentially expressed genes (DEGs), biological processes, and canonical pathways using RNA-sequencing (RNA-seq).

## Materials and methods

### Animals and sample preparation

Adult pregnant female ICR mice at GD 8.5 were purchased from Orient Bio (Gyeonggi-do, Republic of Korea) and had a 7-day refinement period at the Department of laboratory animal resources of Yonsei Biomedical Research Institute. Previous studies have shown that the administration of 100 µg/kg LPS to pregnant mice can induce immune activation along the mother-placenta-fetus axis, which is consequently linked to complicated pregnancy and placental pathology (Kirsten et al. 2013; Fricke et al. 2018; Chen et al. 2016). Exposure of LPS to pregnant mice at concentration of 300–800 µg/kg resulted in severe premature birth and death (Cella et al. 2010; Liu et al. 2016; Zenclussen Ana et al. 2013). Therefore, to establish the conditions that cause defects in growth without being too severe, administration of LPS was carried out at various concentrations. Mice were intraperitoneally administered 200 µl of phosphate-buffered saline (PBS) for control group (n = 6) or varying concentrations of LPS (*Escherichia coli* O127:B8; Sigma Aldrich, St. Louis, MO, USA, L3129) at GD 15.5. The LPS groups were further divided into three groups according to the dose of injected LPS (100, 200, and 400 µg/kg) (n = 6/group). Pregnant mice and embryos were sacrificed 48 h after injection at GD 17.5. After separating the uterus from the dam, the extraembryonic membranes were removed, and embryos were separated from the placentas. All tissues were washed with cold 1× PBS, immediately frozen in liquid nitrogen, and stored at -80 °C until further use. To determine the sex of fetuses, genomic DNA of embryo tail was extracted using a DNA Purification Kit (LaboPass, Seoul, Korea, CMR0112), and PCR was performed using *Sry* primer: F, 5′- CAGCCCTACAGCCACATGAT-3′; R, 5′- GAGTACAGGTGTGCA GCTCTA-3′. Experimental schematic figure for all placenta work is shown in Additional file 1: Fig. S1.

### Pregnancy outcomes

To examine the survival rate, the number of live and dead fetuses was counted, and the ratio was calculated by dividing the number of live fetuses by the total number of fetuses in each group. To examine the growth retardation, the weight and crown rump length of the embryos (n = 70/group) and the weight and length of the placentas (n = 70/group) were measured and categorized according to the sex of the offspring. Embryos showed less than 90% weight of control were classified as IUGR. Placental efficiency was calculated using the following equation: weight of fetus/weight of the placenta ×100.

### Enzyme-linked immunosorbent assay (ELISA)

Maternal serum was collected 4 h after the injection of PBS or 400 µg/kg LPS (n = 2/group). Blood was coagulated at room temperature for 30 min and centrifuged at 2,000 × *g* for 10 min. The supernatant was collected and stored at -20 °C until use. Serum cytokine levels were analyzed for pro-and anti-inflammatory cytokines and chemokines using a Q-Plex™ Custom Kit Mouse (QUANSYS Biosciences, Logan, USA, 107749GR). Tumor necrosis factor-α (TNF-α), Interferon gamma (IFN-γ), Interleukin (IL)-1β, IL-6, IL-10, and chemoattractant (KC) levels were measured.

### Histology

For histological analysis, the whole placentas (n = 10/group) were washed with cold 1× PBS and fixed in 4% paraformaldehyde. Fixed tissues were dehydrated using a graded alcohol series (70, 85, 95, and 100%), cleared with benzene, and embedded in paraffin. The paraffin-embedded tissues were then sectioned into 5-µm-thick slices in a sagittal orientation using a microtome and stained with hematoxylin and eosin. For morphometric analysis, the percentage of surface area in the decidua, junctional zone, and labyrinth to the total placental area was quantified using the Image J software (National Institutes of Health, Bethesda, MD, USA). Representative sections were obtained from the center of each placenta. Analyses of all histological experiments were performed blindly.

### Total RNA isolation and reverse-transcription polymerase chain reaction (RT-PCR)

For total RNA isolation, the whole placentas were ground using pellet pestles (Kimble, London, UK), and the total RNA was extracted using the TRIzol reagent (Invitrogen, Massachusetts, USA, 15,596,018) according to the manufacturer’s instructions. Reverse-transcription was performed with 1 µg of total RNA using ImProm-II Reverse Transcriptase (Promega, Madison, WI, USA, A3803) and RNase inhibitor (Promega, N2515). For quantitative real-time polymerase chain reaction (RT-qPCR), cDNA was amplified using SYBR green PCR Master Mix (Applied Biosystems, California, USA, A46109) on an ABI7300 real-time PCR system (Applied Biosystems) according to the manufacturer’s instructions. All primers used for RT-qPCR are listed in Additional file 2: Table S1. For RT-qPCR validation of RNA-seq, the placentas not used for RNA-seq were used.

### Sample preparation and analysis of total RNA sequencing

For total RNA-seq analysis, total RNAs for four different groups (PBS male, PBS female, LPS male, and LPS female) were prepared. Each RNA sample was pooled using six placentas from two different dams and performed from three biological replicates. The quality of all RNA samples was checked according to RNA integrity number, and all samples exceeded the recommended value, obtained using ND 2000 Spectrophotometer (Thermo Inc., DE, USA). RNA sequencing and data analysis were performed by e-biogen Inc. (Seoul, Korea). Libraries were prepared from total RNA using the NEBNext Ultra II Directional RNA-Seq Kit (NEW ENGLAND BioLabs, Inc., UK). Elimination of rRNA was conducted using the RIBO COP rRNA depletion kit (LEXOGEN, Inc., Austria). The rRNA-depleted RNAs were used for the cDNA synthesis and shearing, and indexing was performed using the Illumina indexes. The enrichment step was performed using PCR. To assess the mean fragment size, libraries were checked using the Agilent 2100 b ioanalyzer (DNA High Sensitivity Kit). Quantification of libraries was performed using the library quantification kit with the StepOne Real Time PCR System (Life Technologies, Inc., USA). High throughput sequencing was conducted as paired end 100 sequencing using Hi seq X10 (Illumina, Inc., USA). Mapping of total RNA-Seq reads were processed using TopHat software (Cole Trapnell et al., 2009.) tool for bam file (alignment file). The alignment files also were used for detecting differential expression of genes, isoforms, and long non-coding RNAs (lncRNAs) using cufflinks. The FPKM (fragments per kilobase of exon per million fragments) was used as the method for determining the expression level of the gene regions. The FPKM data were normalized based on the Quantile normalization method using EdgeR within R (R development Core Team, 2016). To eliminate background signals, FPKM > 1 were further analyzed. DEGs were identified by the criteria p-value < 0.05, and fold change (FC) > 1.5. For global transcriptomic analysis, hierarchical clustering heatmap, Venn diagram, scatter plot, volcano plot, Gene ontology (GO), Kyoto Encyclopedia of Genes and Genomes (KEGG) pathway, and protein-protein interaction (PPI) network were assessed.

### Database

A clustering heatmap was obtained using the MeV software. Analyses of scatter plots and volcano plots were performed using the Excel-based DEG Analysis (ExDEGA) tool. GO and KEGG pathways were analyzed using the online functional annotation tool DAVID (version 6.8; https://david-d.ncifcrf.gov/; accessed on June 18, 2021). Network analysis of PPIs was performed using Cytoscape version 3.7.1. Analysis of lncRNAs was performed using the NONCODE database (http://www.noncode.org/keyword.php).

### Statistical analysis

All data were obtained from three separate experiments and are represented as the mean ± standard error of mean (SEM). A two-way anova and student’s *t*-test were used for comparison between the LPS group and PBS control group in females and males. Differences were considered statistically significant at *p <* 0.05.

## Results

### Mid-gestational maternal LPS exposure induced pregnancy complications

To determine the maternal inflammatory response induced by LPS exposure, the levels of pro- and anti-inflammatory cytokines and chemokines were measured (Additional file 3: Fig. S2). The levels of TNF-α, IFN-γ, IL-1β, IL-6, IL-10, and keratinocyte KC were all increased in the LPS-exposed dams compared with those in the control mice (*p <* 0.05), indicating that LPS administration provoked a maternal immune response. Next, to determine whether maternal LPS administration affects the fetal survival, the survival rate of conceptuses was analyzed by calculating the number of dead and live fetuses (Additional file 4: Fig. S3). We observed significantly decreased proportion of live fetuses at 400 µg/kg LPS group (*p <* 0.01), whereas the other groups showed no significant fetal death or preterm labor.

### Mid-gestational exposure to LPS induced IUGR in a sex-specific manner

**Fig. 1:**
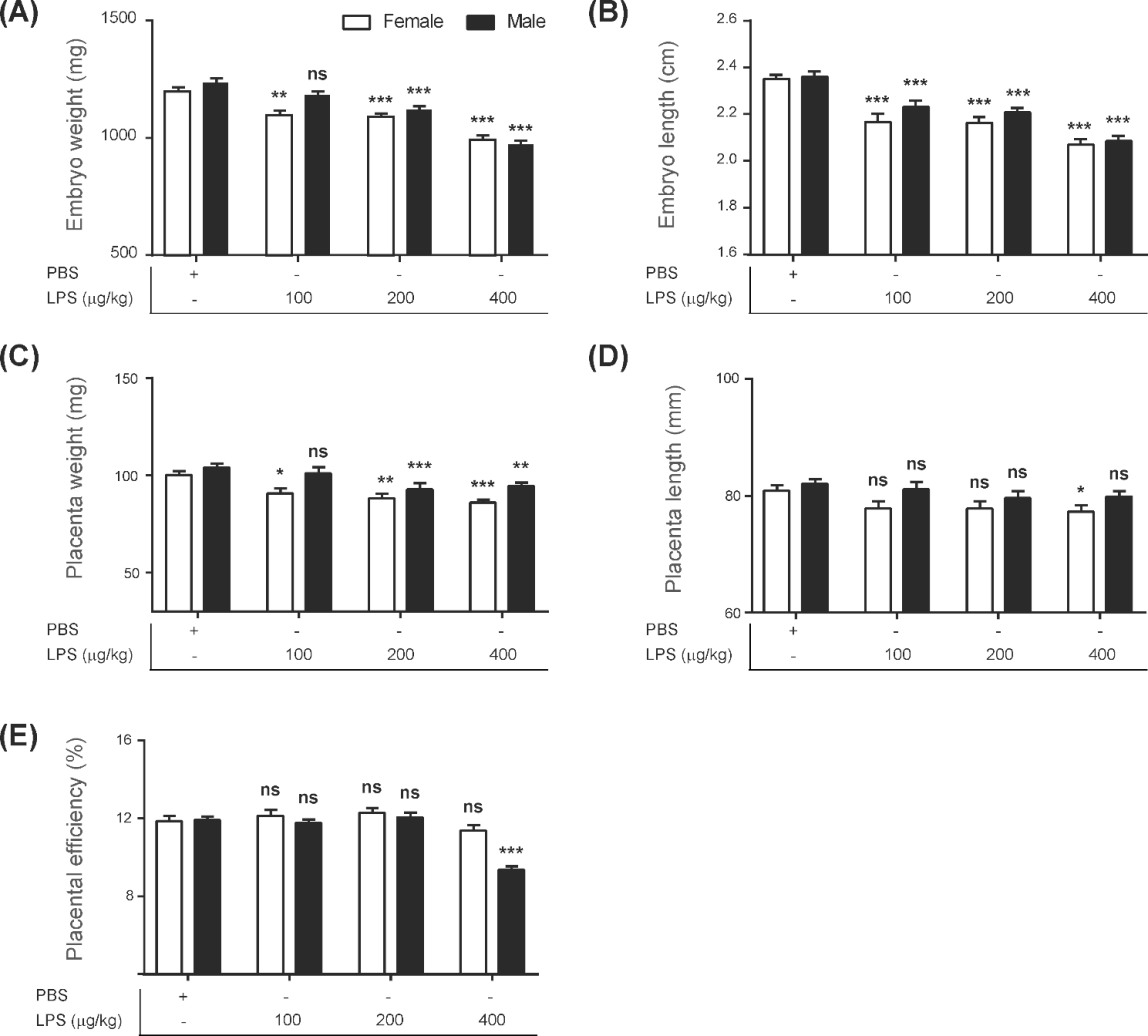
Sexually dimorphic pregnancy complications after prenatal maternal LPS administration At GD17.5, embryos (n = 70/group) and placentas (n = 70/group) treated with PBS or LPS (100, 200, and 400 µg/kg) for 48 h were harvested and analyzed for growth restriction. Embryo weight (**A**) and length (**B**) and placenta weight (**C**) and length (**D**) were measured for female and male. (**E**) Placental efficiency was calculated by dividing the fetal weight by the placental weight. All data were obtained from six dams per group. Differences are represented as the mean ± standard error of mean (SEM). *p < 0.05, **p < 0.01, ***p < 0.001 compared with PBS female or male group using the two-way anova

To evaluate whether prenatal maternal infection leads to abnormal intrauterine fetal growth, we analyzed the weight and length of the embryos following LPS treatment. Exposure of graded LPS to pregnant mice resulted in significantly reduced fetal weight in LPS groups compared with the PBS group (*p <* 0.05, *p <* 0.01) (Fig. 1A). However, IUGR was observed only in 400 µg/kg LPS goup (LPS 100 µg/kg, female; 92%; male; 96%, LPS 200 µg/kg, female; 91%; male; 91%, LPS 400 µg/kg, female; 83%; male; 79%, Additional file 5: Fig. S4A). Surprisingly, the percentage of weight reduction showed a gender difference in that reduction rate was higher in females exposed to 100 µg/kg LPS (*p <* 0.05) and in males exposed to 400 µg/kg LPS (*p <* 0.05), respectively (Additional file 5: Fig. S4A). Administration of graded LPS also reduced length of embryos compared with the control group (*p <* 0.001) (Fig. 1B). However, no statistically significant sex difference was observed in the extent of length decrease in all LPS groups (Additional file 5: Fig. S4B). Placentas of both sexes in the LPS-treated groups showed growth retardation in weight compared with the PBS-treated group (*p <* 0.05, *p <* 0.01, *p <* 0.001) (Fig. 1C). Surprisingly, LPS groups showed more severe decrease in the female placenta (*p <* 0.05) (Additional file 5: Fig. S4C). Moreover, we observed reduction in the length of placenta only in females treated with 400 µg/kg LPS compared with PBS-treated group (*p <* 0.05) (Fig. 1D, Additional file 5: Fig. S4D). Placental efficiency also showed sex-specific alterations, leading to decrease only in 400 µg/kg LPS-treated males compared with PBS, not in females (*p <* 0.001) (Fig. 1E). Collectively, these data indicate that mid-gestational exposure to LPS affects intrauterine growth of fetuses and placentas in a sex-dependent manner.

### Mid-gestational exposure to LPS leads to placental inflammatory condition in a sex-specific manner

**Fig. 2:**
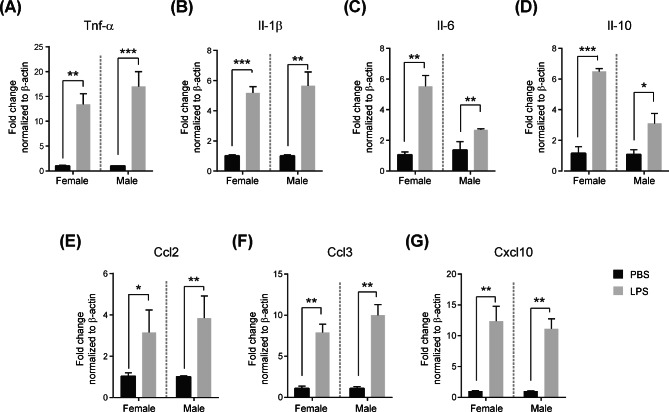
Sexually dimorphic placental inflammation after prenatal maternal LPS infection Results of RT-qPCR analysis of inflammatory cytokines (**A–D**) and chemokines (**E–G**) after administration of LPS (400 µg/kg) for 4 h in female and male placentas. All data were obtained from triplicate experiments, and differences are represented as the mean ± standard error of mean (SEM). *p < 0.05, **p < 0.01, ***p < 0.001 compared with the PBS-treated female or male using the two-way anova

Our results showed the sex-specific placental adaptation at 400 µg/kg LPS, therefore we decided to examine the further studies using the same dose of LPS. To evaluate whether maternal immune activation induced a placental inflammatory response, we analyzed the mRNA levels of placental cytokines and chemokines. Administration of LPS considerably augmented the levels of all cytokines (*p <* 0.05) (Fig. 2A-D) and chemokines (*p <* 0.05) (Fig. 2E-G) in the LPS-exposed placentas compared with the control placentas. Several of LPS-provoked immune signaling molecule genes showed distinction in the degree of increase between females and males. Tnf-α showed a higher rate of increase in males than in (Fig. 2A). Il-6 and Il-10 showed greater sex-biased increase in the female placentas than in males (Fig. 2C and D). Il-1β, C-C motif chemokine ligand (Ccl2), Ccl3, and C-X-C motif chemokine 10 (Cxcl10) did not exhibit clear sex-related differences. These data demonstrate that prenatal maternal LPS exposure results in placental inflammatory condition in a sex-specific manner.

### Mid-gestational exposure to LPS results in impaired placental development in a sex-specific manner

**Fig. 3:**
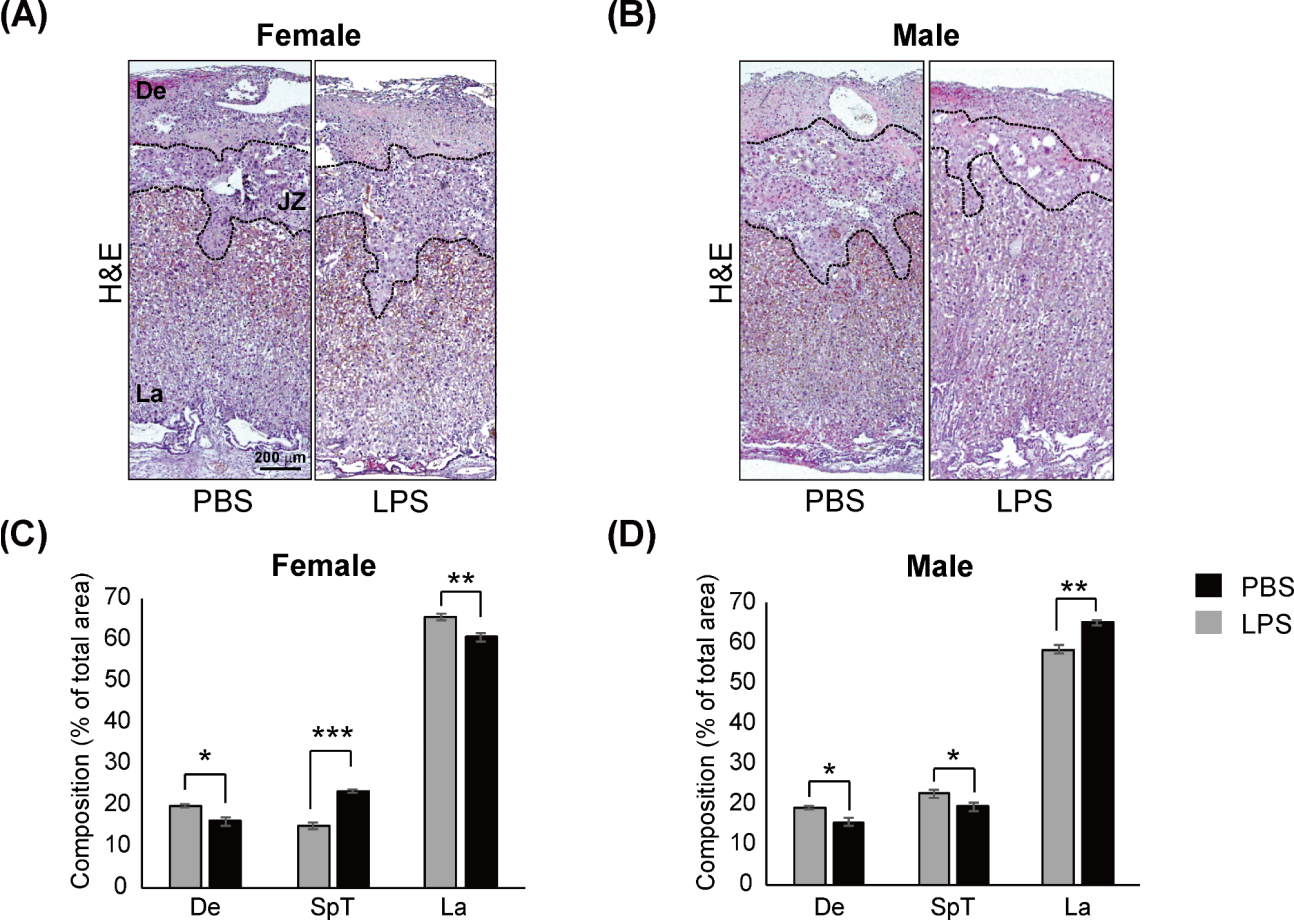
Sexually dimorphic placental pathology after LPS exposure-induced placental inflammation At GD17.5, placentas (n = 10/group) treated with LPS (400 µg/kg) for 48 h were harvested and stained with hematoxylin and eosin. The female placentas (**A**) and male placentas (**B**) were magnified 40x. Scale bar = 200 μm. Black dotted lines divide the trilaminar trophoblast cell layers, and each layer is indicated with black text. Quantitative analysis of the area distribution of (**A**) is shown as bar graph (**C**). Quantitative analysis of the area distribution of (**B**) is shown as bar graph (**D**). The undrawn with black line and uncut data are shown in Additional file 6: Fig. S5. Differences are represented as the mean ± standard error of mean (SEM). *p < 0.5, **p < 0.01, ***p < 0.001 compared with the PBS group using the Student’s t-test. De, decidua; JZ, junctional zone; La, labyrinth

To determine whether maternal LPS administration affected morphogenesis at the fetal–maternal interface, we performed histological analyses by comparing the composition areas of the trilaminar trophoblast cell layers to the total area: decidua (De), junctional zone (JZ), and labyrinth (La) (Fig. 3A, B, Additional file 6: Fig. S5). At GD 17.5, both sexes of the placenta showed decreased percentage area of the decidua in the LPS group compared with that in the control group (*p <* 0.05) (Fig. 3C, D). However, sex-specific alterations were notably observed in the junctional zone and labyrinth layer. In the LPS group, the female placentas showed increased percentage are of junctional zone (*p <* 0.001), whereas the male placentas displayed a reduction in that layer comparison to the PBS group (*p <* 0.05). Additionally, LPS-treated female placentas showed a decreased proportion in the labyrinth layer (*p <* 0.01), whereas LPS-treated male placentas showed an increase compared with that in the control group (*p <* 0.01). These data demonstrate that mid-gestational maternal exposure to LPS induces impaired placental development in a sex-specific manner.

### Mid-gestational exposure to LPS leads to sexually dimorphic features in placental transcriptome profiles

**Fig. 4:**
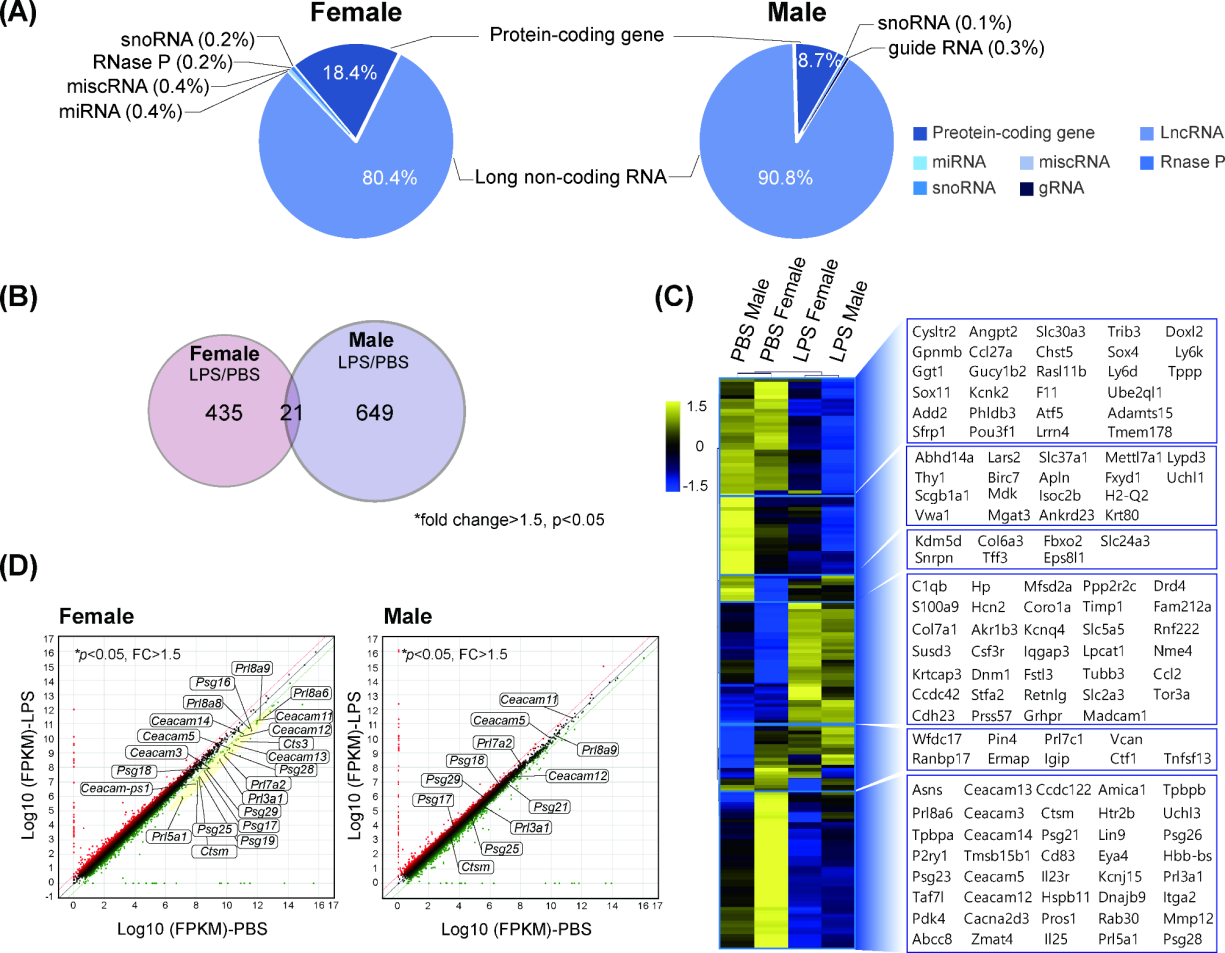
Sexually dimorphic transcriptome analysis of placentas after maternal LPS (400 µg/kg) exposure at mid-gestation (**A**) Distribution of transcript types that are altered differentially in female and male placentas, respectively. (**B**) Venn diagram analysis of differentially expressed genes (DEGs) in LPS/PBS group. (**C**) Hierarchical clustering heatmap and list (right box) of differentially expressed protein-coding genes. (**D**) Scatter plot analysis of DEGs in LPS/PBS group. The clustered gene families that are decreased only in the female placentas are represented by the yellow color in female LPS/PBS group, and the gene symbol is boxed in both sexes. Enrichment of p-value < 0.05 and fold change > 1.5 was considered for DEGs.

We demonstrated that the incidence of prenatal stress-induced placental developmental abnormalities is sex-dependent. However, the mechanisms and genes involved in these phenomena remain unclear. To identify a wide spectrum of sexual dimorphisms under adverse pregnancy conditions, transcriptome analysis was performed using RNA-seq in whole placentas, which were grouped into PBS female/PBS males/LPS female/LPS male. To explore global transcriptomic changes, we analyzed the distribution of the transcript subtypes of DEGs after 400 µg/kg LPS exposure in female and male placentas, respectively (Fig. 4A). Our results showed robust alterations in lncRNA expressions as well as protein-coding genes in both sexes. The number of DEGs in each sex was identified as 456 and 649 genes, respectively (Fig. 4B). The sex-specific DEGs of protein-coding genes are shown using hierarchical clustering and listed according to the expression patterns (Fig. 4C, Additional file 7: Table S2). Hierarchical clustering of representative DEGs of lncRNAs listed in Additional file 8: Table S3 and is shown in Additional file 9: Fig. S6. The representative DEGs that were previously reported to be associated with placental development and pathology are listed in Table 1. Given that our previous study reported the sex-specific regulation of the members of cluster family after prenatal maternal Dexamethasone (DEX) administration (Lee et al., 2017), we expected the alteration in clustered genes. Especially, as shown in the bottom box in Fig. 4C describing the female-specific downregulation, there were changes in clustered gene families, including *pregnancy-specific glycoproteins (Psg)*, *carcinoembryonic antigen-related cell adhesion molecule (Ceacam)*, and *prolactin (Prl)* genes, implicated in immune-related functions during pregnancy. These sex-specific gene regulations are delineated by scatter plot (Fig. 4D, Additional file 7: Table S2). Taken together, our data demonstrate that placental sex affects diverse disparities in transcriptome profiles in response to prenatal maternal stress.

**Table 1:** DEGs previously reported to be associated with placental development and pathology

DEGs in female			
Gene symbol	Related to	Fold change	References (doi)
Tubb3	Preeclampsia	1.632	doi: 10.3390/biology9040062.
Tpbpa	Defects in maternal spiral artery remodeling	0.585	doi: 10.1016/j.ydbio.2011.07.036.
Mmp12	Preeclampsia	0.654	doi: 10.1016/j.placenta.2008.09.015.
Ppp2r2c	Preeclampsia	1.587	doi: 10.1016/j.tjog.2011.07.005.
Cd83	Preeclampsia	0.619	doi: 10.1371/journal.pone.0180065
Hspb11	Preeclampsia	0.593	doi: 10.7150/thno.56141.
Iqgap3	Preeclampsia	1.580	doi: 10.7150/thno.56141.
Drd4	Preeclampsia	1.546	doi: 10.7150/thno.56141.
Krtcap3	Preeclampsia	1.684	doi: 10.14288/1.0074068
1600015I10Rik	Maternal hypoxia	0.311	doi: 10.1177/1933719107302860.
Eya4	Preeclampsia, IUGR	0.582	doi: 10.14288/1.0361940
Kcnk2	Preeclampsia, IUGR	0.538	doi: 10.14288/1.0361940
Susd3	Stillbirths	2.529	doi: 10.1002/pd.2817.
Il23r	IUGR	0.542	doi: 10.1016/j.nutres.2016.08.001.
Vcan	Preeclampsia	0.647	doi: 10.1371/journal.pone.0178549.
Rnf222	Maternal obesity	1.563	doi: 10.1038/s41366-020-0561-3.
Grhpr	Preeclampsia	1.511	doi: 10.3389/fphys.2020.592689.
Slc5a5	Recurrent pregnancy loss/failures	1.571	doi: 10.1159/000508309.
Pdk4	IUGR	0.660	doi: 10.1152/ajpregu.00197.2015.
Cdh23	Preeclampsia	1.576	doi: 10.1371/journal.pone.0222672.
Tpbpb	Gestational Diabetes Mellitu	0.576	doi: 10.1371/journal.pone.0038445.
Kcnk2	Gestational Diabetes Mellitu	0.538	doi: 10.1371/journal.pone.0038445.
Ccl2	IUGR	2.036	doi: 10.1016/j.ajog.2013.03.001.
Slc2a3	IUGR	1.568	doi: 10.1113/JP278473.
Timp1	Preeclampsia	1.792	doi: 10.1095/biolreprod.107.063743.
Lin9	preterm labour	0.646	doi: 10.1186/s12916-016-0632-4.
Sfrp1	preterm birth	0.659	doi: 10.1080/14767058.2017.1359830.
Retnlg	IUGR	2.289	10.1101/2021.03.26.437292
S100a9	IUGR	1.924	10.1101/2021.03.26.437292
Ccl2	Preeclampsia	2.036	doi: 10.3390/biom10060953.
Fbxo2	Preeclampsia, IUGR	1.569	doi: 10.3390/ijms21103597.
Fstl3	Preeclampsia	1.600	doi: 10.1186/s12920-019-0548-x.
Dnm1	Preeclampsia	1.535	doi: 10.1186/1752-0509-6-97.
Tff3	Preeclampsia	1.815	doi: 10.1080/14767058.2021.1888915.
Pros1	Preeclampsia	0.634	doi: 10.3892/ijmm.2012.983.
Uchl3	Preeclampsia	0.662	doi: 10.3892/ijmm.2012.983.
Mfsd2a	Gestational Diabetes Mellitus, Preeclampsia	1.644	doi: 10.3390/nu11051107.
Kcnj15	Preeclampsia	0.566	doi: 10.1038/s41598-020-79008-4.
Kcnq4	Preeclampsia	1.719	doi: 10.1371/journal.pone.0192122.
Ceacam13	IUGR	0.529	doi: 10.1055/s-0029-1224143.
**DEGs in male**			
**Gene symbol**	**Related to**	**Fold change**	**References (doi)**
Krt80	Preeclampsia	0.598	doi: 10.3390/biology9040062.
Fxyd1	preeclampsia	0.347	doi: 10.1016/j.ajog.2010.08.043
Col7a1	IUGR	1.741	doi: 10.1016/j.jri.2010.04.001.
Rasl11b	Preeclampsia	0.632	doi: 10.7150/thno.56141.
Slc37a1	Preeclampsia	0.503	doi: 10.1101/2021.03.11.21253393
1600015I10Rik	Maternal hypoxia	0.330	doi: 10.1177/1933719107302860.
Trib3	Preeclampsia, IUGR	0.578	doi: 10.14288/1.0361940
Birc7	Gestational Diabetes Mellitu	0.633	doi: 10.1016/j.jdiacomp.
Add2	Gestational Diabetes Mellitu	0.591	doi: 10.1016/j.ajog.2008.08.022.
Slc30a3	Preterm birth	0.627	doi: 10.17077/etd.p3zuvpa3
Kcnk2	Gestational Diabetes Mellitu	0.556	doi: 10.1371/journal.pone.0038445.
Ccl2	IUGR	2.105	doi: 10.1016/j.ajog.2013.03.001.
Timp1	Preeclampsia	1.358	doi: 10.1095/biolreprod.107.063743.
Atf5	Early-onset preeclampsia	0.622	doi: 10.1073/pnas.1907548116.
Retnlg	IUGR	2.289	10.1101/2021.03.26.437292
S100a9	IUGR	1.924	10.1101/2021.03.26.437292
Scgb1a1	IUGR	0.623	10.1101/2021.03.26.437292
Ccl2	Preeclampsia	2.105	doi: 10.3390/biom10060953.
Gpnmb	preeclampsia	0.599	doi: 10.1080/14767058.2021.1888915.
Ctf1	preeclampsia	1.547	doi: 10.1074/jbc.M111.230045.
1810011O10Rik	Preeclampsia	0.657	doi: 10.3892/ijmm.2012.983.
Mgat3	Preeclampsia	0.666	doi: 10.3892/ijmm.2012.983.
F11	IUGR	0.586	doi: 10.1038/s41598-018-37627-y.
Angpt2	preeclamsia	0.594	doi: 10.1016/j.placenta.2014.07.001.

### Mid-gestational exposure to LPS affects sexually dimorphic alteration in biological processes and pathways

**Fig. 5:**
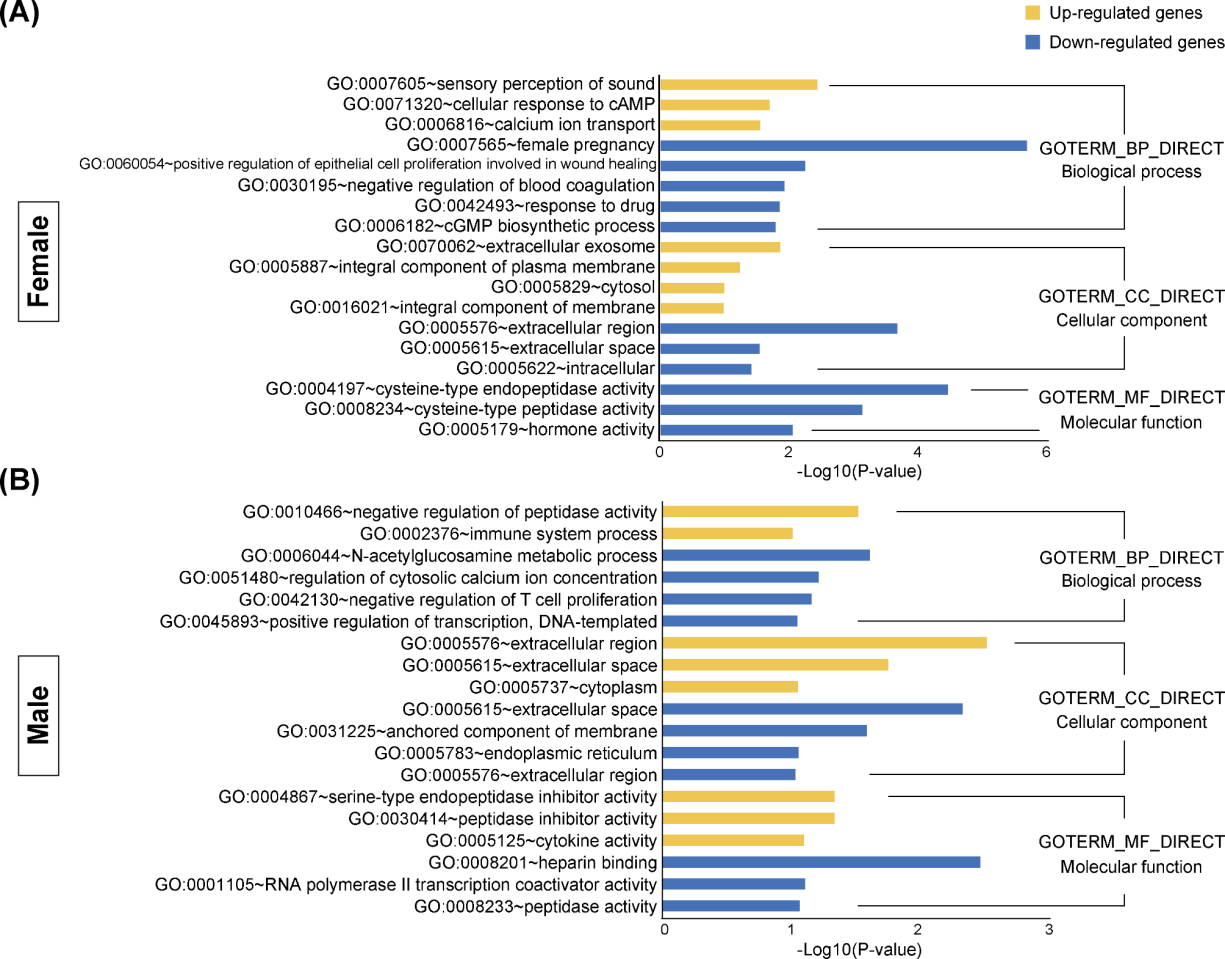
Gene ontology (GO) analysis of differentially expressed genes (DEGs) in the placenta after maternal LPS (400 µg/kg) administration for 48 h GO terms were classified into three categories—biological process, cellular component, and molecular function. GO annotation of upregulated genes (yellow bar) and downregulated genes (blue bar) are shown for female (**A**) and male placentas (**B**). Enrichment of p-value < 0.05 and fold change > 1.5 was considered for DEGs.

**Fig. 6:**
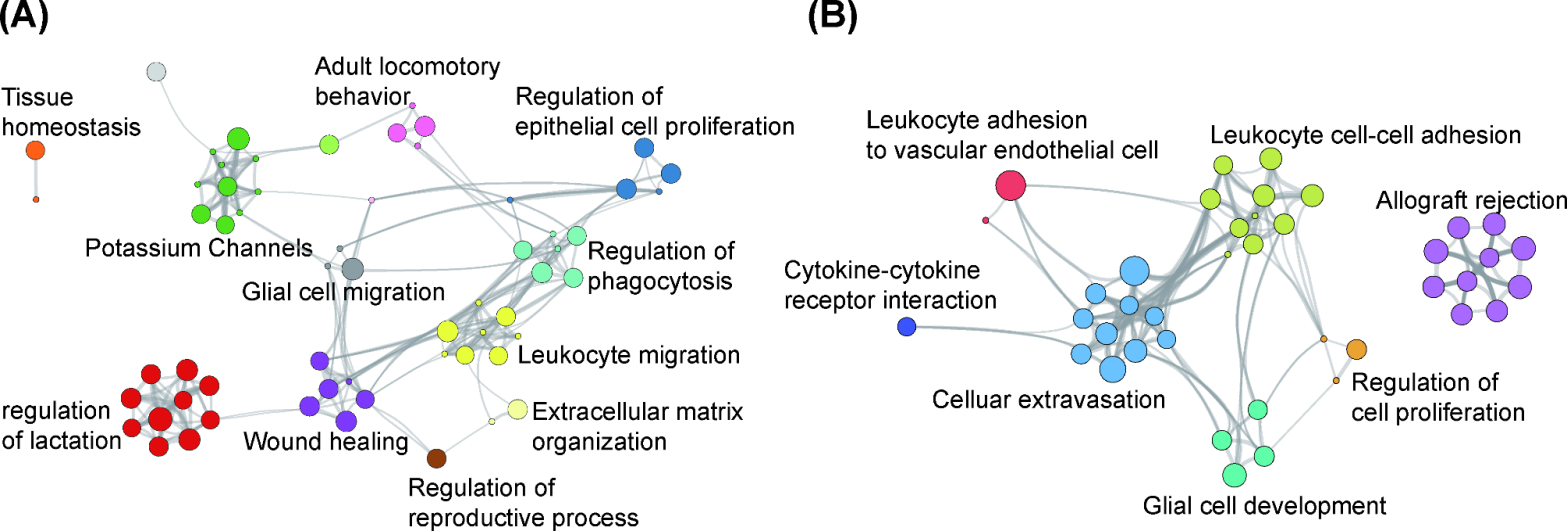
Identifying the protein–protein interaction (PPI) network in placenta after prenatal LPS (400 µg/kg) exposure for 48 h Cytoscape network analysis of differentially expressed genes was performed in female (**A**) and male placentas (**B**). p-value < 0.5 and fold change > 1.5 were used as the cut-off criteria

To gain further insight into key processes that might explain the functional differences between female and male placentas exposed to LPS, DEGs were analyzed using GO analysis. Three categories, including GOTERM_BP_DIRECT for biological processes, GOTERM_CC_DIRECT for cellular components, and GOTERM_MF_DIRECT for molecular function, were assessed. GO enrichments of the DEGs were categorized into 41 and 25 functional groups in LPS-treated female and male placentas, respectively (Fig. 5A, B). In the female placentas, the top five overrepresented pathways for biological processes were implicated in female pregnancy, sensory perception of sound, regulation of epithelial cell proliferation, regulation of blood coagulation, and response to drugs. The male-specific categories showed that regulation of immune system process, T cell proliferation, N-acetylglucosamine metabolic process, peptidase activity, and cytosolic calcium ion concentration were ranked among the top five terms for biological processes. Sexual dimorphism in the potential physiological processes is shown in Table 2. Except for mmu04060: cytokine–cytokine receptor interaction, all other pathways showed non-overlapping pathway enrichment. The representative pathways in the female placentas showed enrichment in mmu04540: gap junction, mmu05142: Chagas disease (American trypanosomiasis), mmu04611: platelet activation, and mmu04080: neuroactive ligand–receptor interaction. In the case of male placentas, mmu04672: intestinal immune network for IgA production, mmu05323: rheumatoid arthritis, mmu04120: ubiquitin mediated proteolysis, and mmu04514: cell adhesion molecules were enriched. To depict the differences in classifications for cellular functions, PPI networks were analyzed, and the results are shown in Fig. 6A and B. In the female placentas, the regulation of reproductive processes, lactation, wound healing, and phagocytosis were identified. However, the male placentas mainly showed interactions associated with immune processes.

**Table 2:** Physiological processes of DEGs in female placentas and male placentas

KEGG pathway for female DEGs	p-value
mmu04540:Gap junction	1.22E + 00
mmu05142:Chagas disease (American trypanosomiasis)	1.08E + 00
mmu04060:Cytokine-cytokine receptor interaction	9.87E-01
mmu04611:Platelet activation	9.04E-01
mmu04080:Neuroactive ligand-receptor interaction	8.32E-01
mmu04145:Phagosome	7.20E-01
mmu05144:Malaria	6.94E-01
mmu05412:Arrhythmogenic right ventricular cardiomyopathy (ARVC)	5.67E-01
mmu04971:Gastric acid secretion	5.41E-01
KEGG pathway for male DEGs	p-value
mmu04060:Cytokine-cytokine receptor interaction	1.69E + 00
mmu04672:Intestinal immune network for IgA production	1.00E + 00
mmu05323:Rheumatoid arthritis	7.34E-01
mmu04120:Ubiquitin mediated proteolysis	5.25E-01
mmu04514:Cell adhesion molecules (CAMs)	4.78E-01
mmu04062:Chemokine signaling pathway	4.11E-01
mmu05168:Herpes simplex infection	3.91E-01

Altogether, these results demonstrate the sex-related differences in placental functional change under adverse *in utero* environments and provide possible different strategies to cope with the increased risks of prenatal complications during pregnancy.

### Mid-gestational exposure to LPS affects the expression of placental genes associated with impaired placental development

**Fig. 7:**
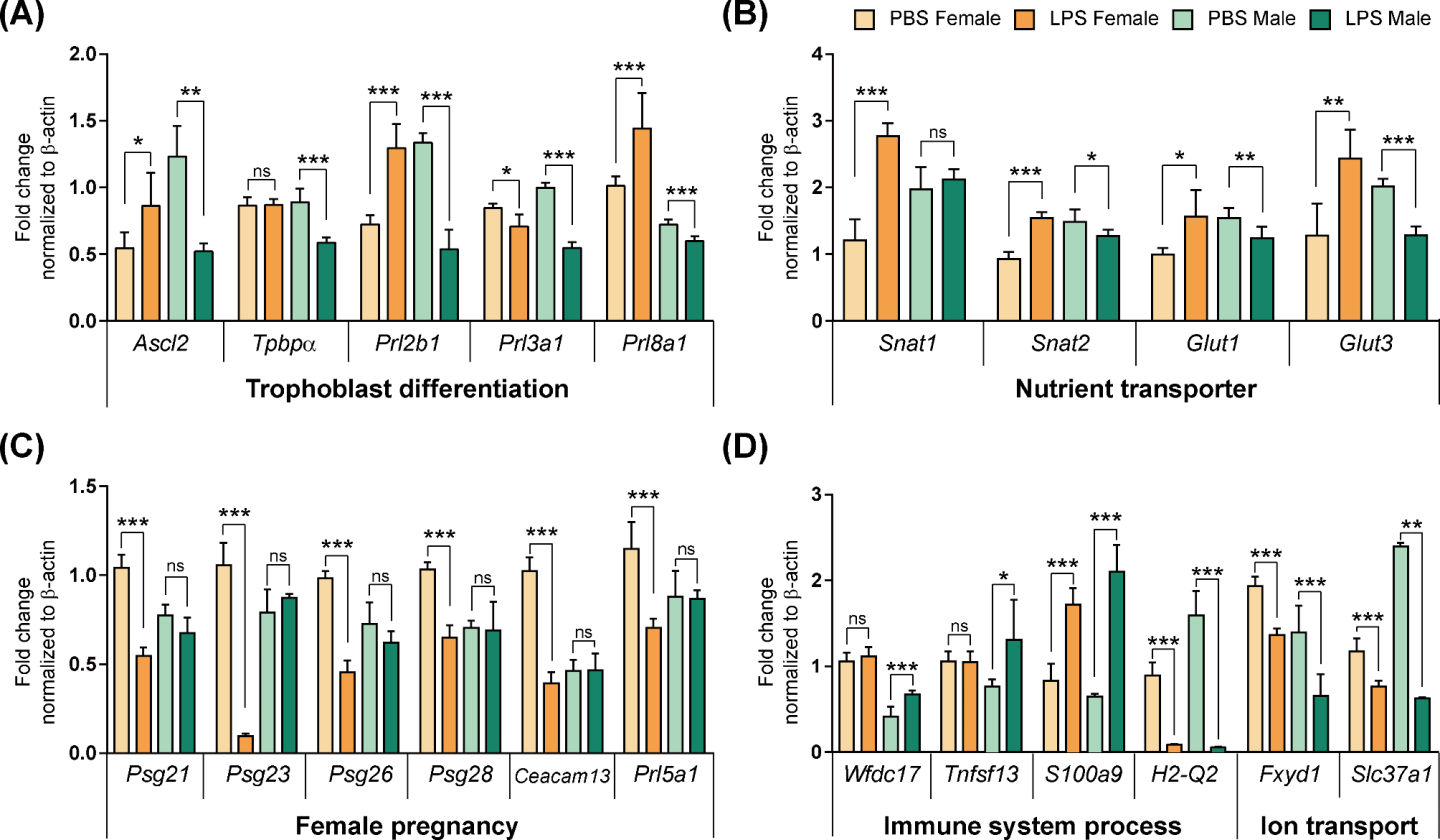
RT-qPCR validation of differentially expressed genes (DEGs) associated with morphological and physiological changes in female and male placentas after maternal LPS (400 µg/kg) exposure for 48 h (**A**) RT-qPCR analysis of DEGs associated with trophoblast differentiation. (**B**) RT-qPCR analysis of DEGs that function as nutrient transporter involved in IUGR. (**C**) RT-qPCR analysis of DEGs categorized as female pregnancy. (**D**) RT-qPCR analysis of DEGs associated with immune system processes and ion transport. All experiments were performed in triplicate. The two-way anova was used for comparison between the PBS and LPS groups in females and males. Differences are represented as the mean ± standard error of mean (SEM). Significant differences were considered as *p < 0.05, **p < 0.01, ***p < 0.001

To validate the transcriptome profiles, we evaluated the expression levels of genes based on RNA-seq results. First, the expression levels of trophoblast differentiation markers were examined in both sexes of placentas in the LPS and PBS groups. The spongiotrophoblast (SpT) and glycogen trophoblast (GlyT) differentiation markers *Ascl2* and *Tpbpα* (SpT and GlyT) were significantly downregulated only in the male placentas (*p <* 0.05, *p <* 0.01) (Fig. 7A). Given that among the Prl family, *Prl2b1*, *Prl3a1*, and *Prl8a1* are markers of SpT cells distinguishable from GlyT and giant cells in the junctional zone (Cross et al.2008*)*, we examined the expression levels of Prl genes. Consistent with the reduction in the junctional zone area, Prl genes were significantly downregulated in the male placentas (*p <* 0.001), whereas *Prl2b1* (P-TGC, SpT, S-TGC) and *Prl8a1* (P-TGC, SpT) were upregulated in the female placentas (*p <* 0.001), indicating concordant results with the junctional zone area. As the ability of the placenta to transport essential nutrients to developing fetuses directly affects fetal growth, we examined the expressions of nutrient transporters. The amino acid transporters, Snat1/*Slc38a1* and Snat2/*Slc38a2*, and the glucose transporters, Glut1/*Slc2a1* and Glut3/*Slc2a3*, were significantly upregulated in the female placentas (*p <* 0.05), whereas they were downregulated in the male placentas (*p <* 0.05) (Fig. 7B).

Next, based on the representative GO terms for each sex, we examined the genes related to female pregnancy for females (Fig. 7C) and the immune system process and ion transport for males (Fig. 7D). *Psg21*, *-23*, *-26*, and *− 28*, *Ceacam13*, and *Prl5a1*, categorized by GO terms for female pregnancy, were downregulated only in the female placentas (*p <* 0.001), not in the male placentas. On the contrary, among the genes corresponding to immune system processes, *Wfdc17* and *Tnfsf13* related to monocyte/macrophage-mediated immunological processes were upregulated only in the male placentas (*p <* 0.05). In addition, there was an increase in *S100a9* (*p <* 0.001) and a decrease in *H2-Q2* expression in male placentas (*p <* 0.001) compared with those in female placentas. *Fxyd1* and *Slc37a1*, which play a role in ion transport, showed a greater decrease in male placentas (*p <* 0.01, *p <* 0.001) than in females.

These data revealed that prenatal maternal LPS stimulation induces sexually dimorphic gene expression signatures in the placenta, providing new evidence for the relationship between sex-biased pathophysiological changes and placental gene expression patterns.

## Discussion

### Prenatal maternal LPS exposure leads to sexually dimorphic placental adaptation with IUGR

**Fig. 8:**
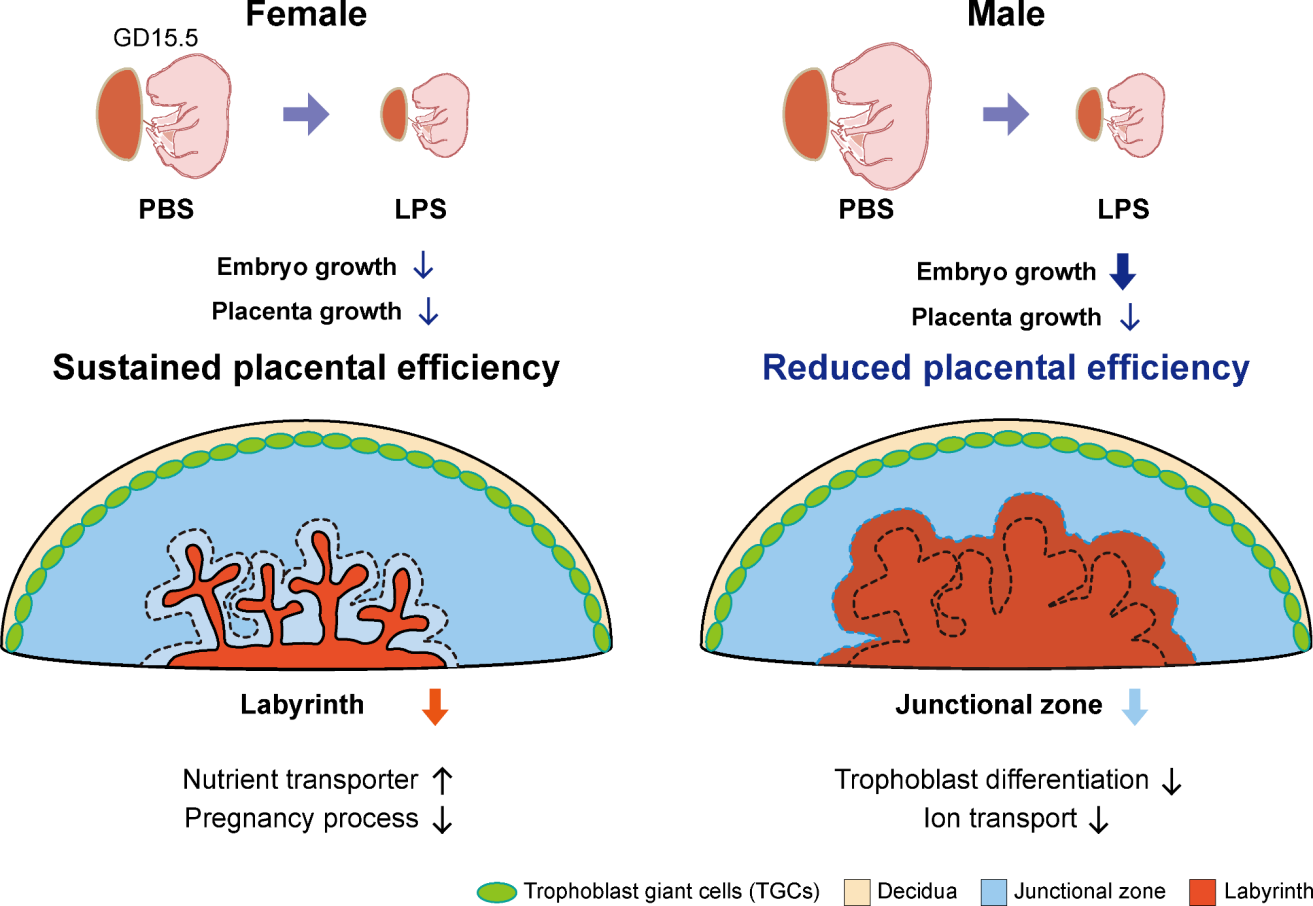
Graphical summary of prenatal maternal LPS (400 µg/kg) exposure-induced sex-specific placental adaptation After 48 h of LPS administration, intrauterine growth restriction of fetuses and placentas were induced in both sexes at the mid-gestational stage. Females showed mild growth reduction in fetuses and placenta, presenting sustained placental efficiency. Males exhibited tremendous reduction in fetuses and mild reduction in placenta growth, indicating reduced placental efficiency under the same *in utero* milieu in which females exist. Sexually dimorphic morphological and physiological changes were induced in the placenta. In the female placenta, distribution of the labyrinth layer was decreased, whereas that of the junctional zone was diminished in male placenta. Disturbances in gene expression were observed in the four GO term categories associated with nutrient transporter, pregnancy process, trophoblast differentiation, and ion transport

Sex-specific differences in fetal growth and survival have been observed in human and rodent studies, suggesting that given that the placenta is an organ mediating intrauterine development, there may be gender differences in placental functions. To date, the increased chance to approach omics, which covers various fields from DNA to protein as well as metabolomics, sheds light on the knowledge that there are sex disparities in DNA methylation, epigenetic modification, gene and protein expression, and immune function in the placenta (Gonzalez et al. 2018; Braun et al. 2021; Liu et al. 2021). Moreover, differences between the sexes in vulnerability to disease, morbidity, and mortality (Stevenson et al. 2000; Renzo et al. 2007; Engel et al. 2008) imply that there are different mechanisms for dealing with intrauterine challenges by sex. In efforts to identify the link between the sex of conceptus and placental adaptations under suboptimal uterine environment, animal models were established by exposure to lead, glucocorticoid, DEX, and alcohol (Sobolewski et al. 2018; Yu et al. 2022; Lee et al. 2017; Loke et al. 2018). In this study, we showed that the administration of graded LPS at the mid-gestational period leads to IUGR and defects in placental development in a dose-dependent manner. In particular, the administration of 400 µg/kg LPS resulted in a sexually dimorphic growth reduction in both embryos and placentas (Fig. 8). In accordance with the signatures of pregnancy complications, disruption in placental structure was induced in a sex-specific manner, in that the female and male placentas showed opposite alteration of the labyrinth layer and junctional zone. Profiling of the placental transcriptome after LPS administration displayed sex-biased gene expression patterns, which contributed to the differences in diverse cellular functions and phenotypic changes observed in both sexes. Therefore, the results of this study demonstrate that the sex of the conceptus exerted potent effects on placental pathophysiology under disturbed *in utero* immune system, highlighting the importance of fetal sex in placental functions and consequent physiological differences that might affect the later life as well as the early life of the fetus by altering fetal programming.

### Sexually dimorphic changes in fetal growth restriction

Animal models established by exposure to prenatal stressors such as mental stress (Bronson and Bale 2014), maternal infection (Stojanovska et al. 2019), high-fat diet (HFD), high-salt diet (Reynolds et al. 2015), and hypoxia (Cuffe et al. 2014) suggest sex differences in pregnancy complications. The maternal asthma model, which characterizes the compromised maternal immune system, demonstrated more still births and lower birth weight in male newborns compared with females (Murphy et al. 2003, 2005). However, chronic asthma leads to IUGR only in male fetuses, which continue to grow normally without any reduction, and females sustained reduced growth with adapted strategies. PE, another common pregnancy-related disorder, is more frequently observed in female fetuses with preterm delivery (< 37 weeks) and in males with delivery at term (37–42 weeks) (Renzo et al. 2007; Vatten and Skjaerven 2004). An animal model established by the administration of LPS and s-Flt1 for PE condition showed that reduced fetal body weight was more prevalent in males, while decreased placental weight was more prevalent in females (Stojanovska et al. 2019; Stark et al. 2009). Likewise, in our models, we observed sustained growth in male fetuses while females showed reduced weight at low dose of LPS. However, the rate of weight reduction tended to be more severe in males receiving high dose of LPS. These observations demonstrate that disturbed immune response by prenatal maternal stress leads to different adversities in growth depending on the sex of the conceptus.

### Sexually dimorphic patterns in placental pathology

Morphological and functional changes within the placenta during the developmental period are crucial to maintain normal fetal growth, and the failure to adapt appropriately to the changes under adverse *in utero* environments is associated with high-risk pregnancy complications (Dahlerup et al. 2018; Meakin et al. 2017; Barry et al. 2008). Animal studies have shown that defects in each layer have region-specific effects on abnormal fetal growth (Woods et al. 2018). Defects in the junctional zone with distribution changes in SpT and the abnormal number and location of GCs were shown to be related to IUGR (Tunster et al. 2016). Targeted gene mutations of *Ascl2*, *Hectd1*, *Htra1*, and *Pdcd5* lead to reduced junctional zones with IUGR, which is caused by a decrease in the number of SpT and GCs, as well as mislocalization of GCs (Oh-McGinnis et al. 2011; Sarkar et al. 2014; Hasan et al. 2015; Li et al. 2017). The manipulation of *Cited1* leads to IUGR, resulting in an enlarged junctional zone and smaller labyrinth (Rodriguez et al. 2004). Impaired labyrinth development and structure lead to defects in the placental ability to transport nutrients to the fetus and consequently IUGR occurs, which is observed in maternal undernutrition, PE, HFD, and inflammation (Gaccioli and Lager 2016; Illsley and Baumann 2020). Previous studies have reported the relevance of the amino acid transporter Snat2 and IUGR in human cohort (Chen et al. 2015a, b) and dysregulation of the glucose transporter Glut1 protein in human PE (Luscher et al. 2017). In this study, we observed the alterations in the junctional zone area and the thickness of the labyrinth and accompanied disturbed gene expression patterns by sex. These results might present a relationship between the sex of the fetus and placental pathophysiology in the IUGR model.

### Sexually dimorphic alteration in placental genes linked to fetal growth and development

Placenta transcriptome studies reported that dysregulated genes during abnormal pregnancy are associated with the processes for regulating fetal growth and development (Sober et al. 2015; Uitert et al. 2015; Majewska et al. 2019; Cox et al. 2015). Here, we observed that female and male placentas showed different gene alteration. Of the DEGs, the members known for their implications in the immune system during gestation (Moldogazieva et al. 2017; Borba et al. 2019) were especially dysregulated in the female placentas, while males showed sustained expression patterns. In particular, as the members of the CEACAM superfamily, CEACAM and PSGs showed sex-dependent expressions. Our previous study showed that prenatal maternal DEX administration induced the dysregulation of PSG gene families in a sex-specific manner (Lee et al. 2017). PRL, prevalently known as a hormone, plays important roles in reproduction and immune response by acting as an inflammatory cytokine (Borba et al. 2019). These genes were shown to be involved in the response to physiological stress in hypoxic placenta and PE (Bu et al. 2016; Lenke et al. 2019). In this study, the PSG, CEACAM, and PRL gene families showed sex-specific expression induced by LPS administration. Disturbed expressions in genes categorized as trophoblast differentiation and nutrient transport has relevance with fetal growth. We observed more severe fetal weight decrease and downregulation of genes, including *Tpbpα, Prl2a1, Prl8a1, Snat2, Glut1*, and *Glut3*, in male placentas compared with than females, indicating the potential association between placental gene expression and fetal growth outcome in a sex-dependent manner.

### Potential roles of lncRNAs in sex-specific placental adaptation

Among the most altered transcripts in both sexes were the lncRNAs, which are implicated in development and diseases of various tissues (Taniue and Akimitsu 2021). The role of lncRNAs in the placenta has been shown to be essential for normal placental development, and is associated with placental diseases accompanied by restricted fetal growth or fetal maldevelopment (Basak and Ain 2019). The association of MALAT1 (metastasis-associated lung adenocarcinoma transcript 1) with pregnancy complications was proposed by revealing dysregulated expression patterns in placenta with PE and increta/percreta (Chen et al. 2015a, b; Tseng et al. 2009). The lncRNA H19 is dysregulated in PE and shown to be involved in regulation of cell proliferation via TGF-β signaling (Zuckerwise et al. 2016). Downregulated H19 is observed in placenta with IUGR, and this transcriptional regulation is mediated by methylation at the promoter region (Koukoura et al. 2012). Studies focusing on epigenetic players exerting sex differences during placental development have proposed DNA methylation as a crucial factor (Martin et al. 2017). According to Mohanty et al. (2015, 2015). Therefore, the robust changes in the expression of lncRNAs in our data are important in itself, and elucidate the potential genes that can affect DNA methylation to mediate other key genes related to placental pathogenesis. Further molecular analysis of unknown lncRNAs in our sequencing data may provide potential clues for the differences between females and males during not only normal pregnancy but also complicated pregnancy caused by defects in placental development.

## Conclusion

In the current study, we find that prenatal maternal LPS infection results in IUGR and presents pathophysiological changes in placenta in a sex-dependent manner. Sexually dimorphic alteration is associated with inflammatory responses, placental growth, composition areas of the trilaminar trophoblast cell layers, and gene expression involved in a variety of biological processes. However, we still face limit that we mainly focused on the association of genes related to GO analysis and the area changes, rather than other potentially important analysis of transcriptome. Therefore, studies with other candidate genes not shown here may provide the possibilities to reveal the uncovered field respect to functional and phenotypic alterations under sub-optimal uterine environment depending on the sex.

### Electronic supplementary material

Below is the link to the electronic supplementary material.

**Supplementary Fig. 1.** Experimental schematic figure of placenta work
All experimental groups were performed by using 6 dams per group. Measurement of the weight and length of the embryos (n = 70/group) and placentas (n = 70/group) were conducted according to the offspring’s sex. For histological analysis, the whole placentas (n = 10/group) were used. For total RNA sequencing, six placentas from two different dams were pooled for each biological replicate. Total RNA-sequencing was performed with two and three biological replicates for the PBS and LPS (400 µg/kg) group, respectively. For qRT-PCR validation, the whole placentas not used for RNA sequencing were selected. Six placentas from two different dams were pooled for each biological replicate. Female and male placentas were obtained from the same dam and paired-compared for total RNA-seq and qRT-PCR validation

**Supplementary Table 1**. Primer sequences for RT-qPCR

**Supplementary Fig. 2.** LPS-induced maternal inflammatory response
Maternal circulatory levels of cytokines and chemokines were measured by ELISA. Serum was collected 4 h after LPS (400 µg/kg) injection

**Supplementary Fig. 3.** Maternal LPS-exposure induced pregnancy complication
Percentage of live embryos was measured from six different dams per group. Differences are represented as the mean ± standard error of mean (SEM). **p < 0.01 compared with control PBS group using the Student’s t-test

**Supplementary Fig. 4.** Prenatal maternal LPS exposure-induced pregnancy complications
At GD17.5, embryos (n = 70/group) and placentas (n = 70/group) treated with LPS (100, 200, and 400 µg/kg) for 48 h were harvested and analyzed for growth restriction. The ratio of alteration in embryo weight (A) and length (B) and placenta weight (C) and length (D) were calculated for female and male. All data were obtained from six dams per group. Differences are represented as the mean ± standard error of mean (SEM). *p < 0.05, **p < 0.01, ***p < 0.001 compared with the PBS-treated female or male using the two-way anova. *p < 0.05 control PBS group using the Student’s t-test

**Supplementary Fig. 5**. Sexually dimorphic placental pathology after LPS exposure-induced placental inflammation
At GD17.5, placentas treated with LPS (400 µg/kg) for 48 h were harvested and stained with hematoxylin and eosin. Female placentas and male placentas were magnified 40x. Scale bar = 200 μm

**Supplementary Table 2**. Differentially expressed genes (DEGs) reaching statistical significance (p-value < 0.05; fold change < 1.5) in female placentas exposed to LPS (400 μg/kg) for 48 h

**Supplementary Table 3**. Differentially expressed lncRNAs reaching statistical significance (p-value < 0.05; fold change < 1.5) in female placentas exposed to LPS (400 μg/kg) for 48 h

**Supplementary Fig. 6**. Sexually dimorphic transcriptome analysis of placentas after maternal LPS exposure at mid-gestation
(A) Hierarchical clustering heatmap of differentially expressed lncRNAs. Venn diagram analysis (B) and volcano plot (C) of differentially expressed lncRNAs in LPS/PBS group. Enrichment of p-value < 0.05 and fold change > 1.5 was considered for DEGs.

## Data Availability

All data are available in the main text or the supplementary materials. The results of placenta RNA-seq data have been deposited in GEO database under the accession number GSE182462.

## List of Abbreviations

PBS: Phosphate-buffered saline
LPS: Lipopolysaccharide
GD: Gestational day
RNA-seq: RNA-sequencing
RT-qPCR: Quantitative real-time polymerase chain reaction
DEGs: Differentially expressed genes
IUGR: Intrauterine growth restriction
ASD: Autism spectrum disorder
PE: Preeclampsia
HFD: High-fat diet
ICR: Institute of Cancer Research
ELISA: Enzyme-linked immunosorbent assay
TNF-α: Tumor necrosis factor-α
IFN-γ: Interferon gamma
IL-1β: Interleukin 1 beta
IL-6: Interleukin 6
IL-10: Interleukin 10
CCL2: Chemokine ligand 2
CCL3: Chemokine ligand 3
CXCL10: C-X-C motif chemokine 10
lncRNAs: Long non-coding RNAs
FPKM: Fragments per kilobase of exon per million fragments
FC: Fold change
GO: Gene ontology
KEGG: Kyoto encyclopedia of genes and genomes
PPI: Protein-protein interaction
SEM: Standard error of mean
De: Decidua
JZ: Junctional zone
La: Labyrinth
SpT: Spongiotrophoblast
GlyT: Glycogen trophoblast
GC: Glycogen
Psg: Pregnancy-specific glycoproteins
Ceacam: Carcinoembryonic antigen-related cell adhesion molecule
Prl: Prolactin
