# Supplementary figures and images for: Regulation of sexually dimorphic placental adaptation in LPS exposure-induced intrauterine growth restriction
Source: Mol Med. 2023 Sep 18;29:114. doi: 10.1186/s10020-023-00688-5 (PMC10506314; doi:10.1186/s10020-023-00688-5)

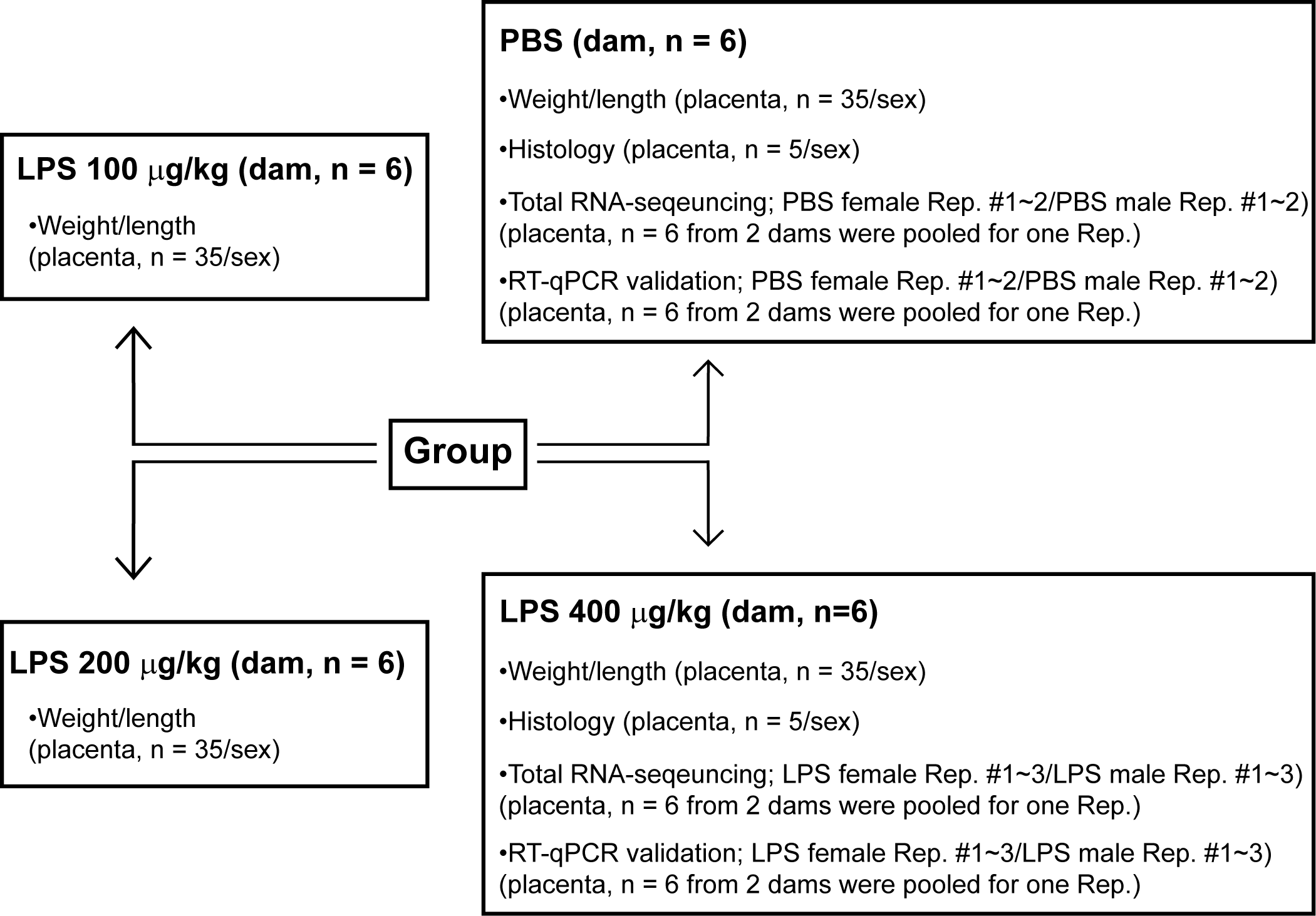

Supplement: Supplementary file 1 — Supplementary Fig. 1. Experimental schematic figure of placenta work All experimental groups were performed by using 6 dams per group. Measurement of the weight and length of the embryos (n = 70/group) and placentas (n = 70/group) were conducted according to the offspring’s sex. For histological analysis, the whole placentas (n = 10/group) were used. For total RNA sequencing, six placentas from two different dams were pooled for each biological replicate. Total RNA-sequencing was performed with two and three biological replicates for the PBS and LPS (400 µg/kg) group, respectively. For qRT-PCR validation, the whole placentas not used for RNA sequencing were selected. Six placentas from two different dams were pooled for each biological replicate. Female and male placentas were obtained from the same dam and paired-compared for total RNA-seq and qRT-PCR validation [file 10020_2023_688_MOESM1_ESM.tif]

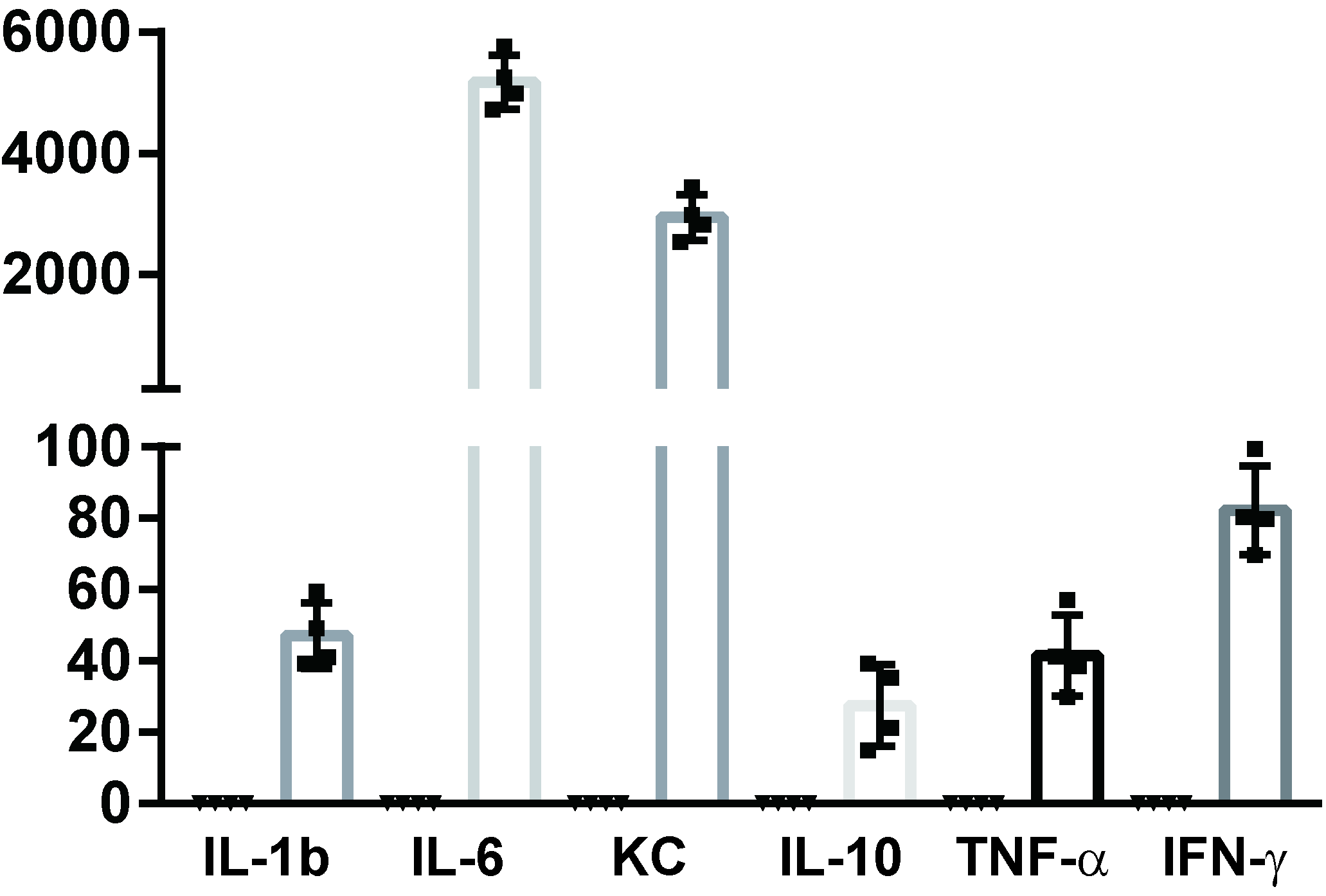

Supplement: Supplementary file 3 — Supplementary Fig. 2. LPS-induced maternal inflammatory response Maternal circulatory levels of cytokines and chemokines were measured by ELISA. Serum was collected 4 h after LPS (400 µg/kg) injection [file 10020_2023_688_MOESM3_ESM.tif]

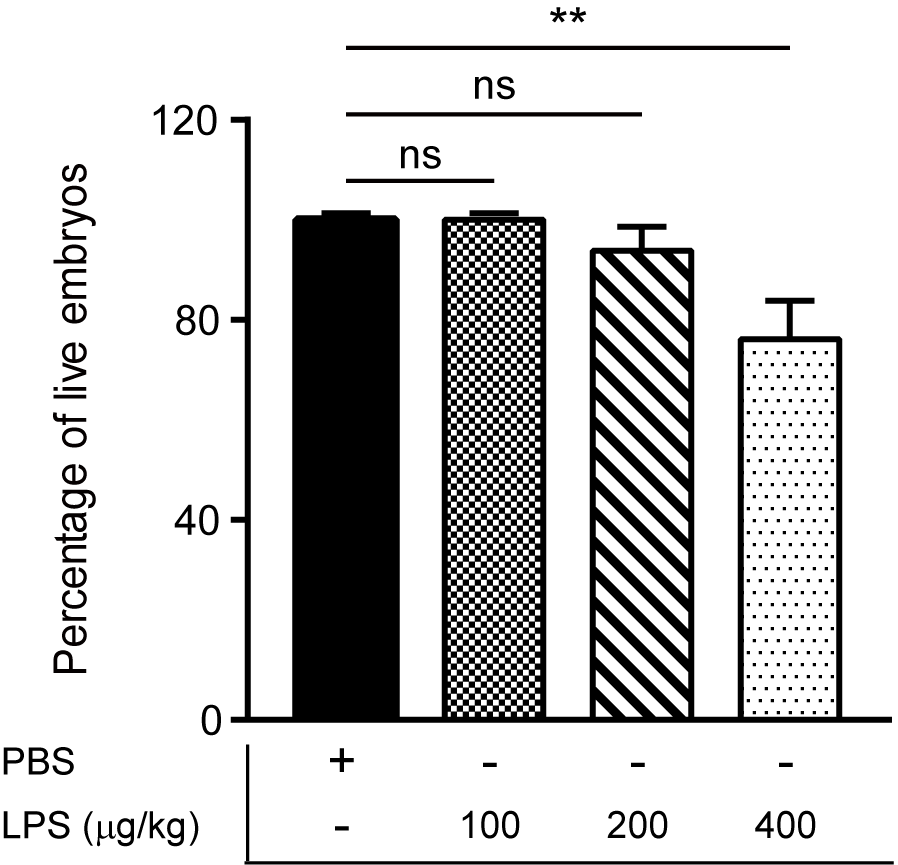

Supplement: Supplementary file 4 — Supplementary Fig. 3. Maternal LPS-exposure induced pregnancy complication Percentage of live embryos was measured from six different dams per group. Differences are represented as the mean ± standard error of mean (SEM). **p < 0.01 compared with control PBS group using the Student’s t-test [file 10020_2023_688_MOESM4_ESM.tif]

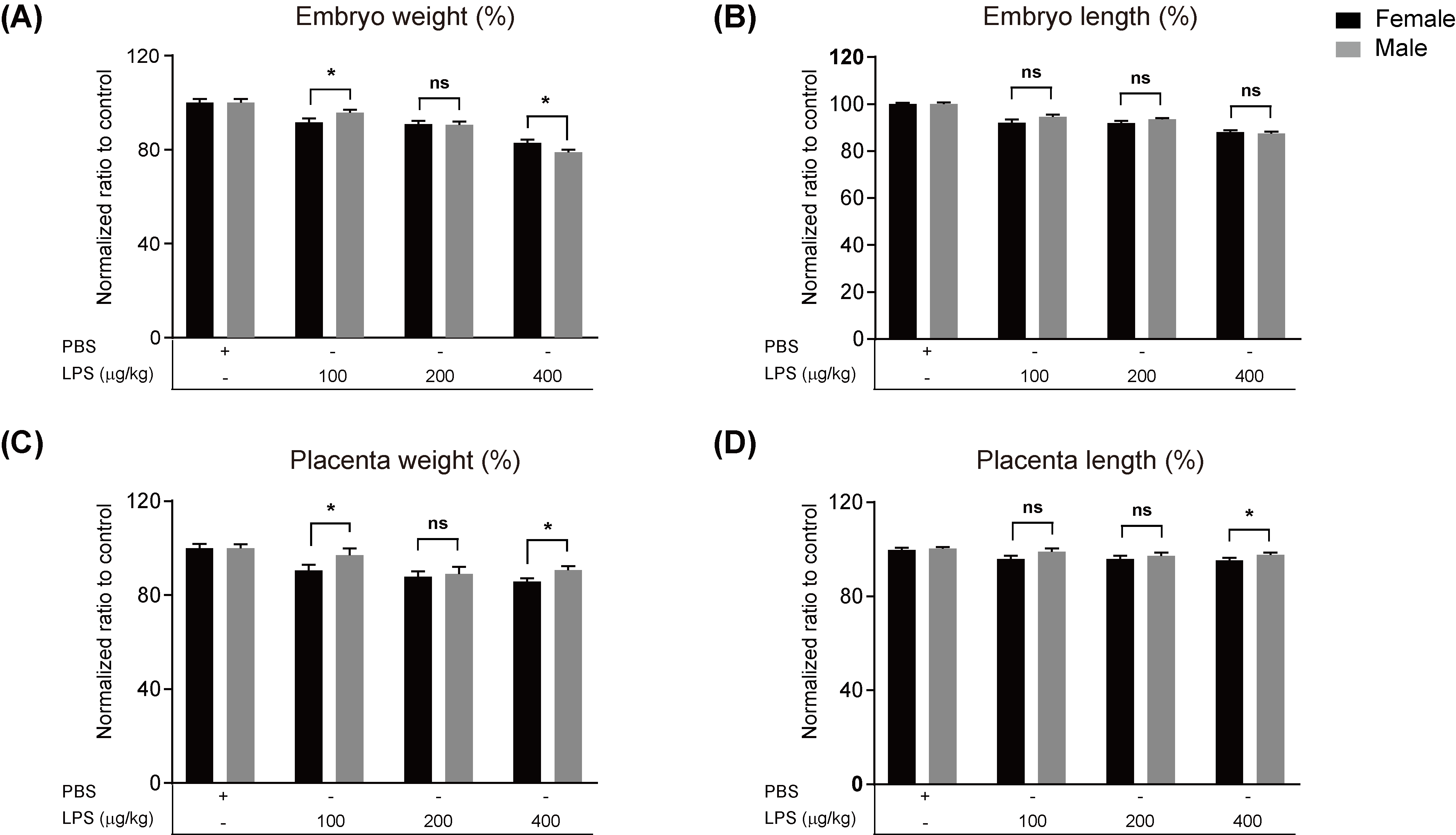

Supplement: Supplementary file 5 — Supplementary Fig. 4. Prenatal maternal LPS exposure-induced pregnancy complications At GD17.5, embryos (n = 70/group) and placentas (n = 70/group) treated with LPS (100, 200, and 400 µg/kg) for 48 h were harvested and analyzed for growth restriction. The ratio of alteration in embryo weight (A) and length (B) and placenta weight (C) and length (D) were calculated for female and male. All data were obtained from six dams per group. Differences are represented as the mean ± standard error of mean (SEM). *p < 0.05, **p < 0.01, ***p < 0.001 compared with the PBS-treated female or male using the two-way anova. *p < 0.05 control PBS group using the Student’s t-test [file 10020_2023_688_MOESM5_ESM.tif]

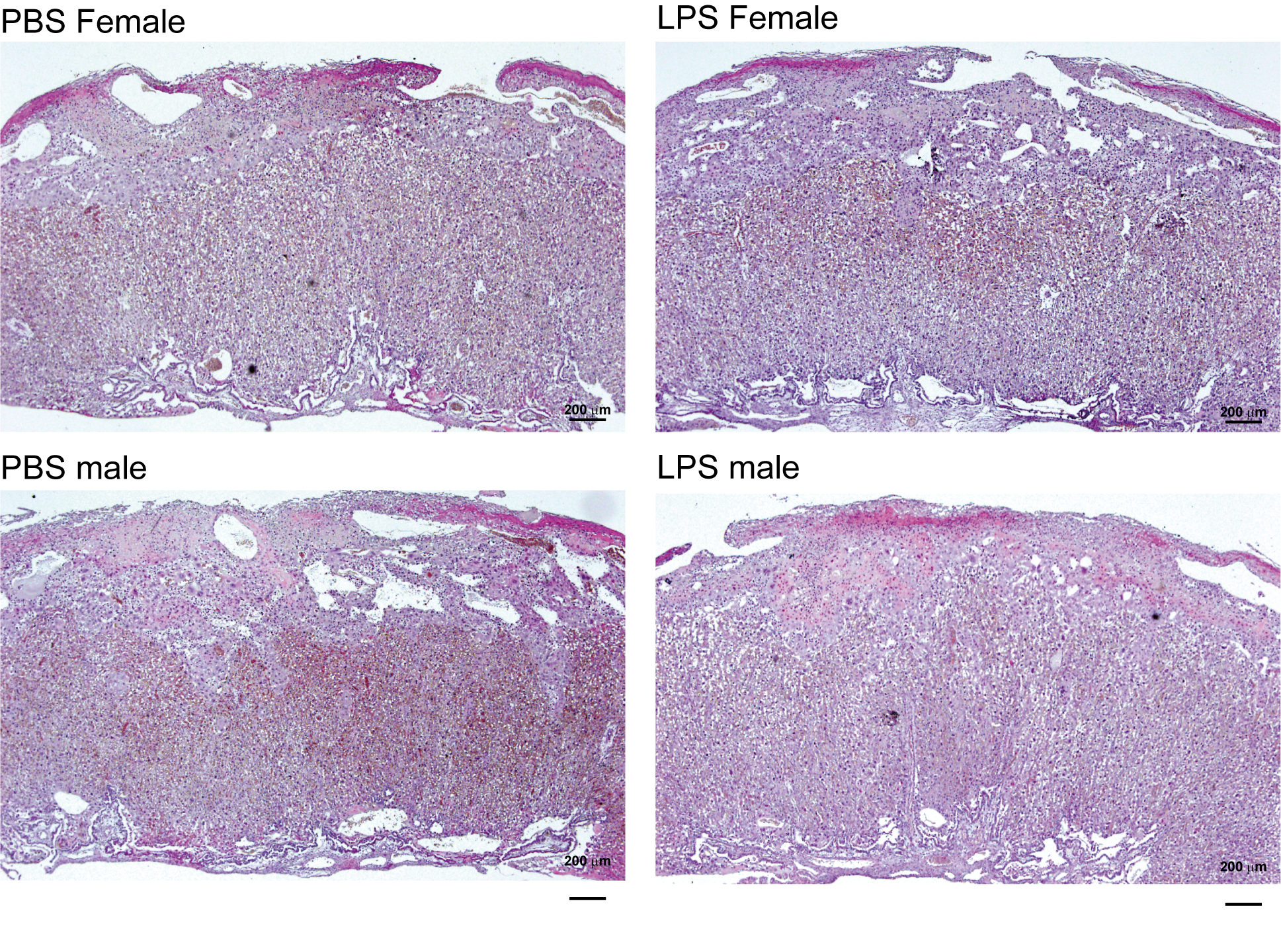

Supplement: Supplementary file 6 — Supplementary Fig. 5. Sexually dimorphic placental pathology after LPS exposure-induced placental inflammation At GD17.5, placentas treated with LPS (400 µg/kg) for 48 h were harvested and stained with hematoxylin and eosin. Female placentas and male placentas were magnified 40x. Scale bar = 200 μm [file 10020_2023_688_MOESM6_ESM.tif]

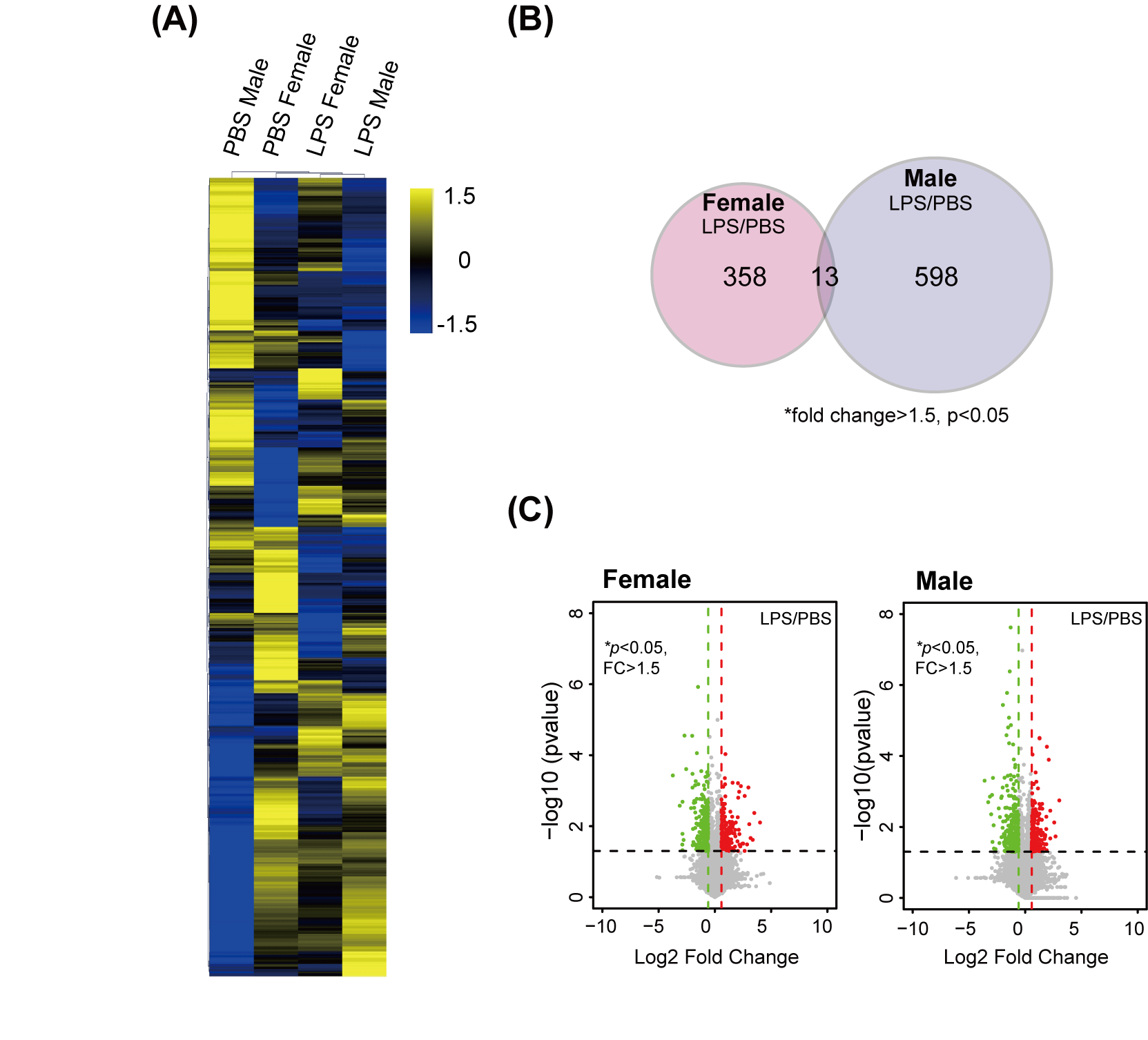

Supplement: Supplementary file 9 — Supplementary Fig. 6. Sexually dimorphic transcriptome analysis of placentas after maternal LPS exposure at mid-gestation (A) Hierarchical clustering heatmap of differentially expressed lncRNAs. Venn diagram analysis (B) and volcano plot (C) of differentially expressed lncRNAs in LPS/PBS group. Enrichment of p-value < 0.05 and fold change > 1.5 was considered for DEGs. [file 10020_2023_688_MOESM9_ESM.tif]
