# Supplementary material for: Regulation of sexually dimorphic placental adaptation in LPS exposure-induced intrauterine growth restriction
Source: Mol Med. 2023 Sep 18;29:114. doi: 10.1186/s10020-023-00688-5 (PMC10506314; doi:10.1186/s10020-023-00688-5)
Supplement: Supplementary file 2 — Supplementary Table 1. Primer sequences for RT-qPCR [file 10020_2023_688_MOESM2_ESM.docx]

Supplementary Table 1. Primer sequences for RT-qPCR

|  | Forward primer (5’-3’) | Reverse primer (5’-3’) |
| --- | --- | --- |
| Tnf-a | TAGCCCACGTCGTAGCAAAC | TTCTCCAGCTGGAAGACTCC |
| Il-1b | GAAGAAGAGCCCATCCTCTGT | TTGTCGTTGCTTGGTTCTCC |
| Il-6 | GAGAGTAGTGAGGAACAAGCC | GCTTCGTCAGCAGGCTGGC |
| Il-10 | GCGCTGTCATCGATTTCTC | TGGAGTCCAGCAGACTCAAT |
| Ccl2 | TCCCTGTCATGCTTCTGGG | GCTTGGTGACAAAAACTACAGC |
| Ccl3 | CCCAGCCAGGTGTCATTTTC | TCCAAGACTCTCAGGCATTCAG |
| Cxcl10 | ACTCAAGGGATCCCTCTCG | CTTGATGGTCTTAGATTCCGG |
| Ascl2 | CTCTGTCCTGCGCCTCTAC | ACCCAGGGATGCAGCTTAG |
| Tpbpa | CAGGTACTTGAGACATGACTC | GGCAGAGATTTCTTAGACAATG |
| Prl2b1 | CGAACGCTCTGAATCATCTT | CTCTCTCCAATCATGCTTTG |
| Prl3a1 | GGGTTCATCCTGGAGCTGAA | TCTGGATAACCAAGGACCAA |
| Prl5a1 | ACCACACCAATCAGGGACAC | GCAGCCAGCATTCTAATTGT |
| Prl8a1 | CAAGAGTTTCTCCACAATGAGT | GACATCATTCATGGCACTCA |
| Glut1/Slc2a1 | ACCTATGGCCAAGGACACAC | GGAGTGTCCGTGTCTTCAGC |
| Glut3/Slc2a3 | GCCTTGGCTCTGCTACACAC | ACCTCACACCTTTCCATTGC |
| Slc37a1 | CATGGGGATAGTGTGCTTTC | GCAGGGAGGACAACAATATG |
| Snat1/Slc38a1 | TCTATGCAGCAGAGGCTCAC | ATTTTGGGCACGTCTGTTTC |
| Snat2/Slc38a2 | CATAGGCAAGGTATGTCTGC | AAGCTTTGGAATTTGGCCTG |
| Psg21 | CAGTGCATACAGCCGAAGAG | CTGTGCTGTCCGTGACTCTC |
| Psg23 | TTCGCCGTCACAAGATGGCG | TTAGGAGCAAGCGAGCAGGC |
| Psg26 | TTCTGCTCACAGCCTCCCTC | CAGCCCCTTCACCGTTACAC |
| Psg28 | ACTTGCACGTGTACTCCTCTC | TCTCTACCACTGTGGGCAG |
| Ceacam13 | ACTCTGCTGCACTTTCCTGT | CATTCTTTGGCAGGTTATGC |
| Wfdc17 | AGAGCCAACATGAAGACAGC | GGAGTTTTGCAGACATGACC |
| Tnfsf13 | CTGCTACAGTGCAGGTGTCT | GGCTGTGTGGGAGATAAACA |
| S100a9 | CTTTAGCCTTGAGCAAGAAG | TTGCCATCAGCATCATACAC |
| H2-Q2 | AGGGAGGCTCTCACACTCTC | ACCTTGAGAACTGGGGTGAT |
| Fxyd1 | ATTACCACACCCTGCGGA | GAAAGTTCCCTCCTCTTCGT |
| β-actin | CATGTTTGAGACCTTCAACACCCC | GCCATCTCCTGCTCGAAGTCTAG |

**Abbreviations:** RT-qPCR, quantitative real-time polymerase chain reaction.
